# Inhibition of NOS- like activity in maize alters the expression of genes involved in H_2_O_2_ scavenging and glycine betaine biosynthesis

**DOI:** 10.1038/s41598-018-31131-z

**Published:** 2018-08-22

**Authors:** Kyle Phillips, Anelisa Majola, Arun Gokul, Marshall Keyster, Ndiko Ludidi, Ifeanyi Egbichi

**Affiliations:** 1 Plant Biotechnology Research Group, Department of Biotechnology, University of the Western Cape, Robert Sobukwe Road, Bellville, 7530 South Africa; 2 Environmental Biotechnology Laboratory, Department of Biotechnology, University of the Western Cape, Robert Sobukwe Road, Bellville, 7530 South Africa; 3 Centre of Excellence in Food Security, University of the Western Cape, Robert Sobukwe Road, Bellville, 7530 South Africa; 4 Department of Biological and Environmental Sciences, Walter Sisulu University, Nelson Mandela Drive, Mthatha, 5117 South Africa

## Abstract

Nitric oxide synthase-like activity contributes to the production of nitric oxide in plants, which controls plant responses to stress. This study investigates if changes in ascorbate peroxidase enzymatic activity and glycine betaine content in response to inhibition of nitric oxide synthase-like activity are associated with transcriptional regulation by analyzing transcript levels of genes (betaine aldehyde dehydrogenase) involved in glycine betaine biosynthesis and those encoding antioxidant enzymes (ascorbate peroxidase and catalase) in leaves of maize seedlings treated with an inhibitor of nitric oxide synthase-like activity. In seedlings treated with a nitric oxide synthase inhibitor, transcript levels of betaine aldehyde dehydrogenase were decreased. In plants treated with the nitric oxide synthase inhibitor, the transcript levels of ascorbate peroxidase-encoding genes were down-regulated. We thus conclude that inhibition of nitric oxide synthase-like activity suppresses the expression of ascorbate peroxidase and betaine aldehyde dehydrogenase genes in maize leaves. Furthermore, catalase activity was suppressed in leaves of plants treated with nitric oxide synthase inhibitor; and this corresponded with the suppression of the expression of catalase genes. We further conclude that inhibition of nitric oxide synthase-like activity, which suppresses ascorbate peroxidase and catalase enzymatic activities, results in increased H_2_O_2_ content.

## Introduction

Nitric oxide (NO˙) is a signaling molecule which regulates biochemical, metabolic and physiological processes that are beneficial to plants^1–3^. Apart from the aforementioned role, there are several lines of evidence supporting the role of NO˙ in the scavenging of reactive oxygen species which are a consequence of normal biological processes and during biotic or abiotic stress in plant tissues^4–6^. This beneficial role of NO˙ in plants is in part due to regulation of antioxidant enzymes such as ascorbate peroxidase (APX) and catalase (CAT) and regulation of the levels of compatible solutes such as glycine betaine^7,8^.

One of the proposed enzymatic sources of NO˙ is the nitric oxide synthase (NOS)-like enzymatic reaction which converts L-arginine into L-citrulline, with simultaneous release of NO˙^9,10^. NOS-like activities sensitive to mammalian NOS inhibitors were initially detected in plant extracts^11^ and are now acknowledged as important sources of NO˙^12^. Various other sources of NO˙ exist in plants; including nitrate reductase (NR), nitrite-NO reductase (NiNOR), xanthine oxidoreductase (XOR) and some non-enzymatic reactions^12^. Given the fact that the use of Nω-Nitro-L-Arginine methyl ester (L-NAME) to inhibit NO˙ biosynthesis has routinely been undertaken to study the physiological functions of NO˙ in plants, including studies on plant development and enzymatic activities that regulate various metabolic activities^12–14^, this approach provides a convenient tool to study NO˙-mediated signaling processes in plants.

We previously showed that application of L-NAME (an analogue of L-arginine, which functions as a competitive inhibitor of animal NOS-mediated NO˙ synthesis) significantly decreased NO˙ content, which resulted in a reduction of the content of glycine betaine (GB) in maize roots and leaves^14^. Furthermore, this NOS inhibition was associated with decreased activity of APX. A reversal of these effects was observed when plants treated with the NOS inhibitor were supplemented with the NO˙ donor 2, 2′-(hydroxynitrosohydrazono)bis-ethanimine (DETA-N0)^14^. However, in view of the fact that CAT is also involved in the catalytic detoxification of excess H_2_O_2_ in plant cells^15^, it is important to investigate if inhibition of NOS-like activity may influence CAT enzymatic activity.

Furthermore, the requirement for NOS-like activity in the regulation of APX activity^14^ may be due to regulation of the expression of genes encoding APX (which has not been explored in maize leaves to date). This would be in addition to well-documented post-translational modifications which activate APX by S-nitrosylation in the presence of NO˙^16,17^ or inactivated in the presence of excessive NO˙-derived peroxynitrite due to tyrosine nitration^17^. Various lines of evidence suggest the existence of at least eight genes encoding APX in maize^18^. APX1.1 occurs in chromosome 1 and its protein is predicted to localize in the cytosol, APX 2 and APX6 occur on chromosome 2 with the APX2 protein predicted to localize in the cytosol whereas the APX6 protein is predicted to localize in mitochondria^18^. APX4 is located in chromosome 4 and the encoded protein is predicted to localize in the cytosol, APX 7 occurs on chromosome 5 with a predicted protein localization in the chloroplast whereas APX1.2 occurs in chromosome 9 and its protein is predicted to localize in the cytosol^18^. APX3 and APX5 occur on chromosome 10, with the APX3 protein predicted to localize in peroxisomes whereas the APX5 protein is predicted to localize in mitochondria^18^.

GB occurs in maize leaves, although in low quantity under normal conditions^7,19^. It is synthesized from its precursor (choline) in a two-step oxidation reaction, through the intermediate betaine aldehyde. The first oxidation step is catalyzed by choline monooxygenase (CMO, EC 1.14.15.7), with further oxidation to GB being catalyzed by betaine aldehyde dehydrogenase (BADH, EC 1.2.1.81)^20^. An increase in BADH activity and elevated GB content occurs in plants under abiotic stress^21–23^. This suggests that the key determining enzyme in the biosynthesis of GB accumulation is BADH.

Several studies have analyzed the transcripts of genes encoding BADH, CAT and APX in plants under various abiotic stress^21,24,25^. To our knowledge, particularly in maize, there have been no studies investigating the expression of the genes involved in GB production in the absence of stress to evaluate the effect of perturbed NO˙ content and in relation to the antioxidant enzymes involved in the regulation of H_2_O_2_ content in plant tissue. We thus investigated the effect of application of L-NAME to maize seedlings in terms of the levels of NO˙ and H_2_O_2_ in maize leaves. We have also analyzed the expression of genes involved in H_2_O_2_ scavenging (namely APX and CA), together with those involved in GB biosynthesis (namely BADH) upon treatment of maize seedlings with L-NAME. Furthermore, we investigated the effect of inhibition of NOS-like activity on CAT enzymatic activity.

## Results

### Nitric oxide and H_2_O_2_ content in *Zea mays* leaves

**Figure 1:**
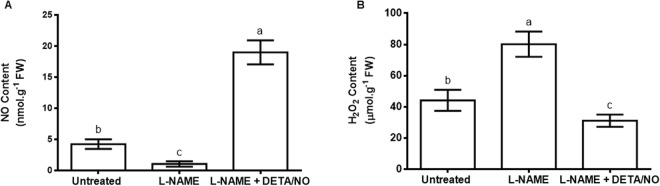
Nitric oxide content in maize leaves in response to inhibition of nitric oxide synthase-like activity by L-NAME (**A**). Levels of H_2_O_2_ in maize leaves in response to treatment with L-NAME (**B**). For both (**A**,**B**), DETA/NO was used as a NO donor to offset the effect of L-NAME on nitric oxide content. Error bars represent means ± S.D. (n = 3). Different letters above bars indicate statistically significant differences between means of the treatments (*p* ≤ 0.05).

Application of L-NAME caused a decrease in NO˙ content in maize leaves. NO˙ content decreased 0.4 times in leaves when compared to the NO˙ content of leaves from the untreated (control) maize (Fig. 1A). The decrease in NO˙ content as a result of treatment with L-NAME was reversed in response to a combination of L-NAME and DETA/NO and was in fact approximately 3 times higher than the untreated control (Fig. 1A). Treatment with L-NAME resulted in a significant increase in leaf H_2_O_2_ content in comparison to the untreated control (Fig. 1B). However, supplementing the L-NAME treatment with DETA/NO reversed the increase observed in the ‘L-NAME only’ treatment in respect of H_2_O_2_ levels (Fig. 1B).

### NOS-like inhibition influences the expression of ascorbate peroxidase

**Figure 2:**
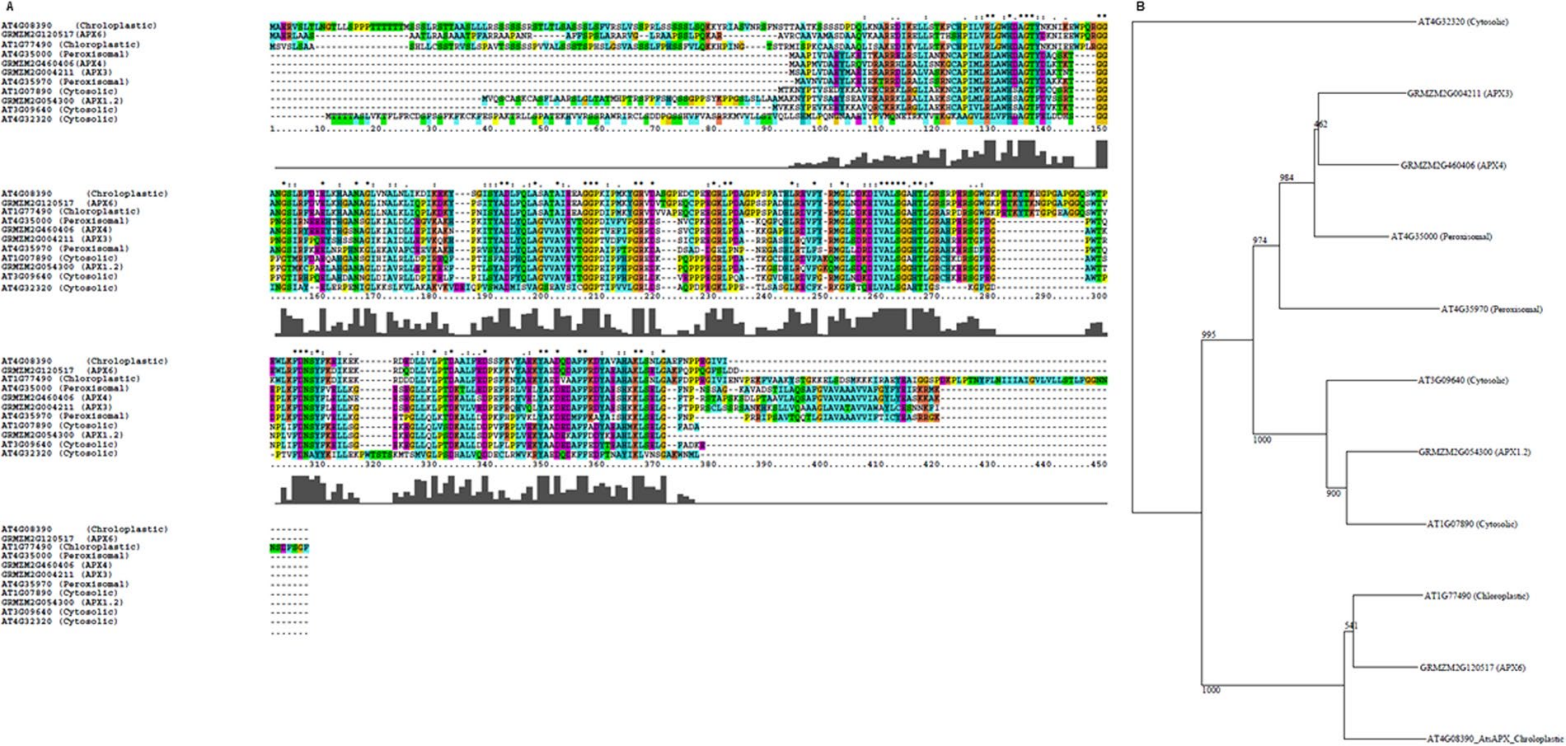
Alignment of maize and Arabidopsis ascorbate peroxidases to illustrate similarity of the maize proteins to the Arabidopsis proteins (**A**). Neighbor Joining tree, generated from the alignment, to group maize ascorbate peroxidases with corresponding Arabidopsis proteins (**B**).

**Figure 3:**
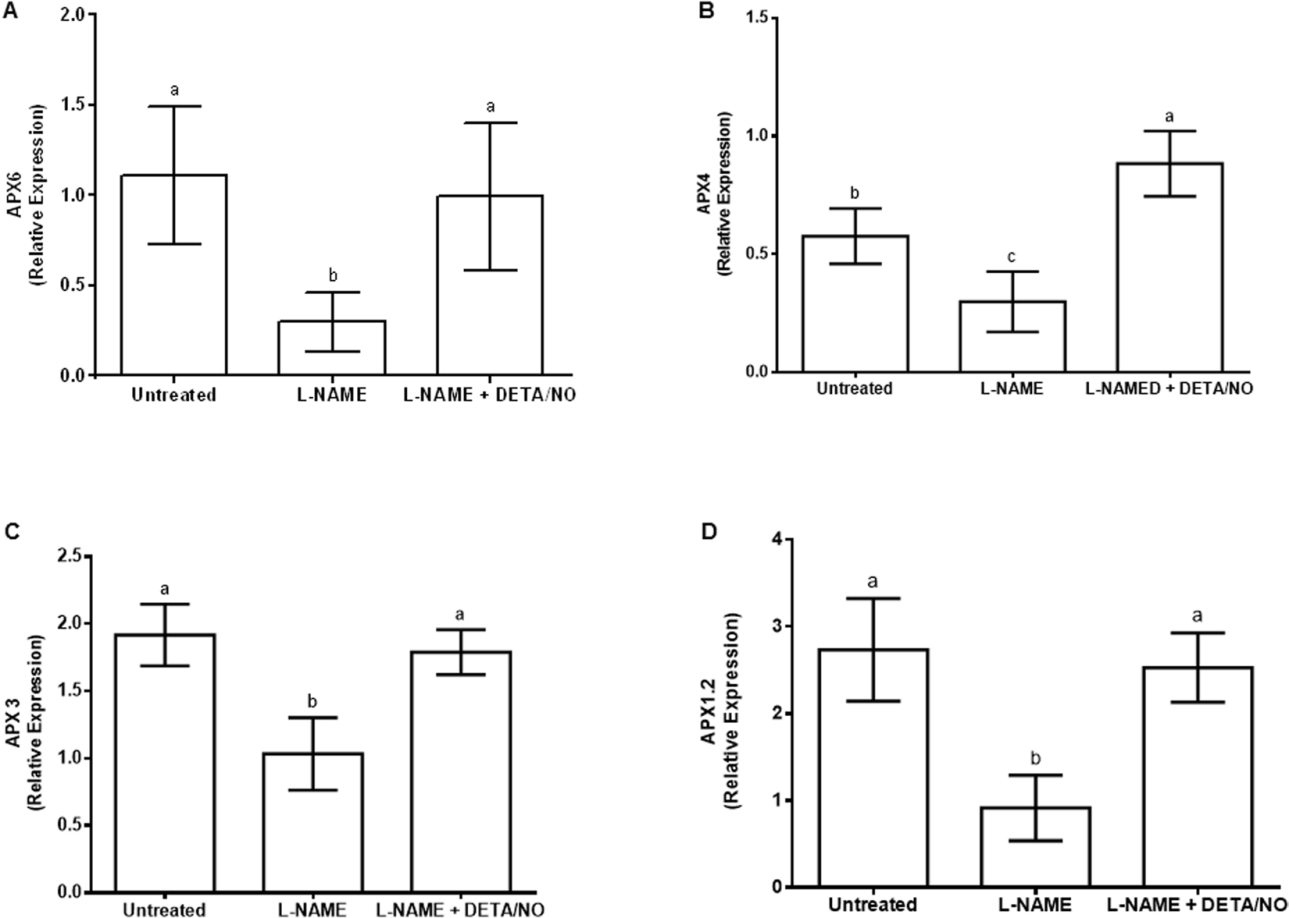
Expression of maize ascorbate peroxidase (APX) genes APX6 (**A**), APX4 (**B**), APX3 (**C**) and APX1.2 (**D**) in maize leaves in response to inhibition of nitric oxide synthase-like activity by L-NAME. Transcript levels are expressed relative to the average level of expression of three reference genes (Elongation Factor 1α, β-tubulin and Actin 2) in each treatment. In all cases, DETA/NO was used as a NO donor to offset the effect of L-NAME on nitric oxide content. Error bars represent means ± S.D. from three independent experiments. Different letters above bars indicate statistically significant differences between means of the treatments (*p* ≤ 0.05).

Sequence analyses showed that maize APX proteins encoded by four APX genes shared sequence similarity with Arabidopsis APX proteins that have previously been analyzed (Fig. 2A). Comparison of both the alignment and phylogenetic tree generated from the alignment showed that the predicted localization of the APX-encoding genes, based on the localization of the Arabidopsis homologues to maize, was either peroxisomal, cytosolic or chloroplastic (Fig. 2A,B). Quantitative RT-PCR showed that application of L-NAME down-regulated the expression of the four ascorbate peroxidase genes investigated in this study. L-NAME decreased the expression of APX6 by 0.6 times, APX4 by 0.4 times, APX3 by 0.44 times and APX1.2 by 0.6 times, respectively, when compared to untreated control (Fig. 3A–D). The decrease in APX gene expression was reversed to the same level of expression as the untreated control when L-NAME and DETA/NO were applied. Furthermore, the expression of APX4 in response to a combination of L-NAME and DETA/NO was 0.35 times higher than the untreated control (Fig. 3A–D).

### BADH genes are down-regulated in response to inhibition of NOS-like activity

**Figure 4:**
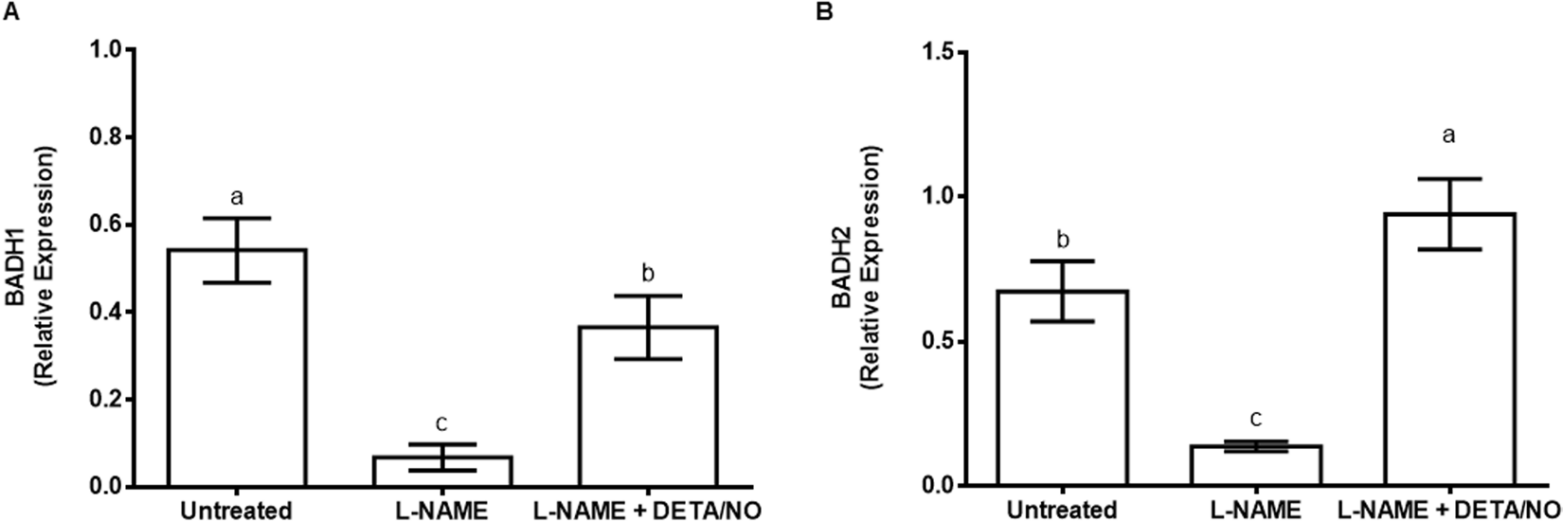
Transcript levels of maize betaine aldehyde dehydrogenase (BADH) genes BADH1 (**A**) and BADH2 (**B**) in maize leaves in response to treatment with L-NAME. Expression levels are relative to the average level of expression of three reference genes (Elongation Factor 1α, β-tubulin and Actin 2) in each treatment. For both (**A**,**B**), DETA/NO was used to offset the effect of L-NAME on nitric oxide content. Error bars represent means ± S.D. (n = 3). Different letters above bars indicate statistically significant differences between means (*p* ≤ 0.05).

L-NAME decreased the expression of BADH1 0.85 times and BADH2 0.8 times respectively when compared to untreated control (Fig. 4A,B). The expression of BADH genes was differentially regulated when plants were treated with a combination of L-NAME and DETA/NO. Whereas BADH1 was down-regulated (0.3 times lower than the untreated control) in response to the combination of L-NAME and DETA/NO, the expression of BADH2 in response to the same treatment was 0.3 times higher than the untreated control (Fig. 4A,B).

### Effect of inhibition of NOS activity on catalase enzymatic activity

**Figure 5:**
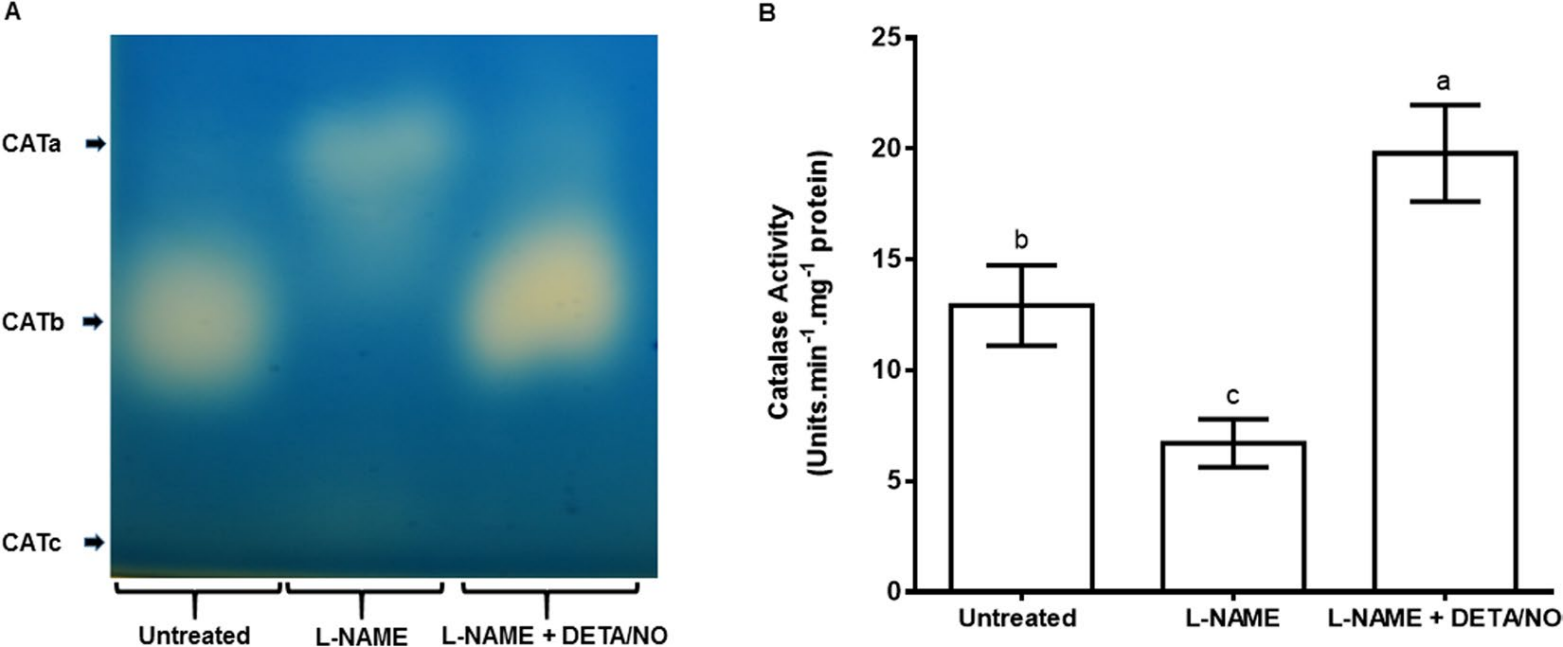
Enzymatic activity of catalase (CAT) as determined by native PAGE (**A**) and spectrophotometry (**B**) in maize leaves in response to treatment with L-NAME. In both cases, DETA/NO was used to offset the effect of L-NAME on nitric oxide content. Error bars represent means ± S.D. from three independent experiments, where bars with different letters signify statistically significant differences between means (*p* ≤ 0.05).

Analyses of the response of CAT enzymatic activity to treatment with L-NAME in maize leaves was carried out using a native PAGE enzymatic assay. Visual assessment showed that the ‘L-NAME’ treatment resulted in two isoforms, which we name CATa and CATc, named according to their migratory pattern, which were not visible in both the untreated control and the combined treatment (L-NAME + DETA/NO). However, CATb from the ‘L-NAME + DETA/NO’ combined treatment had the highest activity as evidenced by its high band intensity and was not detected in the ‘L-NAME’ treatment (Fig. 5A).

There was a pronounced decrease in total CAT activity in response to L-NAME, as determined using spectrophotometry (Fig. 5B). Application of L-NAME decreased total CAT activity by approximately by 0.53 times when compared to untreated control (Fig. 5B). On the other hand, a treatment in which L-NAME was combined with DETA/NO resulted in increased CAT activity (by approximately 0.46 times) when compared to the untreated control (Fig. 5B).

### Inhibition of NOS-like activity influences the expression of CAT1 and CAT2

**Figure 6:**
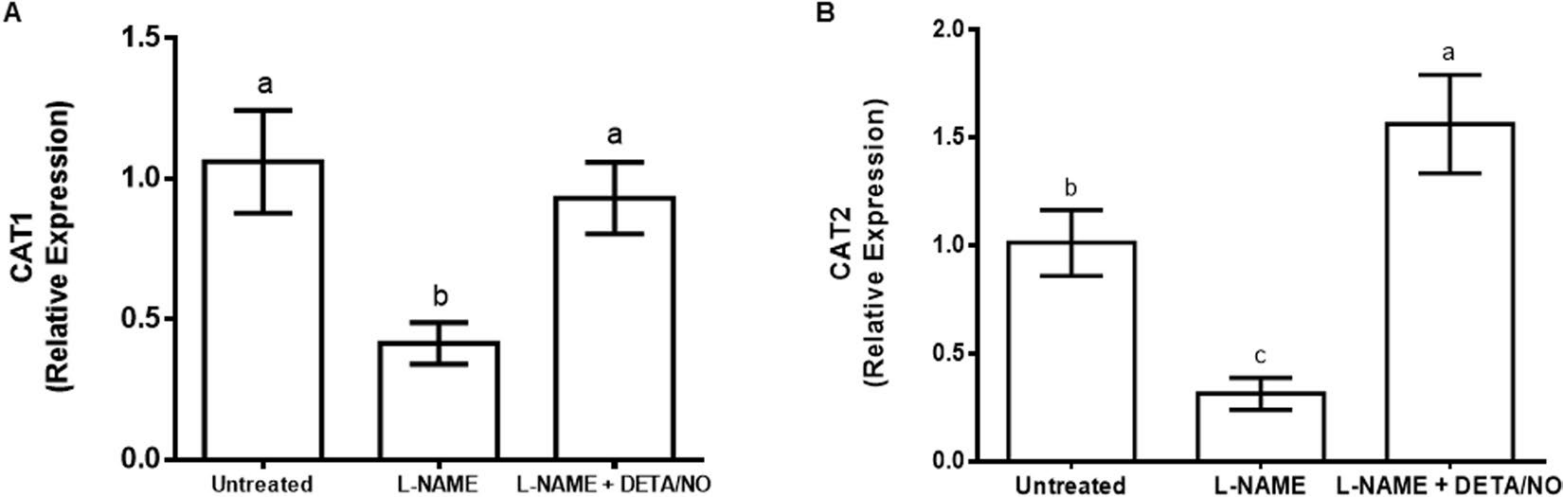
Expression of maize betaine catalase (CAT) genes CAT1 (**A**) and CAT2 (**B**) in maize leaves in response to treatment with L-NAME. Transcript levels are relative to the average level of expression of three reference genes (Elongation Factor 1α, β-tubulin and Actin 2) in each treatment. In botch cases, DETA/NO was used to offset the effect of L-NAME on nitric oxide content. Error bars represent means ± S.D. obtained from three independent experiments. Different letters above bars indicate statistically significant differences between means (*p* ≤ 0.05).

The expression of CAT1 and CAT2 in leaves from plants treated with L-NAME were 0.6 and 0.65 times less than the untreated control, respectively (Fig. 6A,B). However, whereas the expression of CAT1 was reversed to the same level of expression as the untreated control in plants treated with the combination of L-NAME and DETA/NO, the expression of CAT2 in response to the combination treatment was 0.6 times higher than the untreated control (Fig. 6A,B).

## Discussion

The role of NO˙ in maintaining normal plant growth and development has been described previously^26,27^, regulating stomatal closure and photosynthesis^28,29^. Furthermore, NO˙ is a key signaling molecule that mediates responses to abiotic stress^30–32^. We have previously shown that inhibition of NOS-like activity in maize leaves decreases APX activity and glycine betaine content^14^. The results reported in this current study shows that L-NAME decreases NO˙ content and this decrease is reversed by addition of the NO˙ donor DETA/NO. This shows that the changes in NO˙ content observed in our study are a result of perturbation of NOS-like enzymatic activity. We chose an L-NAME concentration of 1 mM and a DETA/NO concentration of 200 µM in this study because these are the concentrations which have previously been shown to lead to pronounced decrease in NO˙ in response to L-NAME and almost complete reversal of the effect of L-NAME by exogenously applied DETA/NO in soybean^13^. At these concentrations, L-NAME causes an increase in H_2_O_2_ content in maize and this is reversed in plants where the L-NAME treatment is supplemented with DETA/NO, which suggests that NO˙ derived from NOS-like activity is required for maintaining H_2_O_2_ levels within the homeostatic redox range. This supports previous reports that show that NO˙ participates in the regulation of ROS levels in plants^33^, particularly H_2_O_2_ levels in the case of this study.

NO˙ has been shown to protect plant tissue against oxidative stress through up-regulation of the activity of antioxidant enzymes^34^. Furthermore, NOS-like activities identified in several plant species in the absence or presence of conditions in which ROS levels change^9,35,36^ suggest that the regulation of ROS levels in plants involve participation of NOS-like activity. The fact that the scavenging of one of these ROS, namely H_2_O_2_, is associated with suppression of APX enzymatic activity in response to inhibition of NOS-like activity in maize leaves^14^ presents a possibility that NOS-like activity may regulate the expression of APX genes in maize leaves. In this study, it is clear that inhibition of NOS-like activity leads to a pronounced decrease in the expression of at least four maize APX genes. This shows that, besides the already known post-translational modification of APX proteins by S-nitrosylation^16,17^, NO˙ derived from NOS-like activity in maize is required for the expression of APX-encoding genes. This demonstrates that both the transcriptional and post-translational regulation of APX are influenced by NO˙. It will be useful in future to identify *cis*-acting elements associated with NO˙-mediated transcriptional regulation of the APX genes in maize, not only limited to simple bioinformatics prediction but directed to *in vivo* studies which analyze the effect of serial deletions in the promoters of the APX genes to pin-point DNA-protein interactions that influence NO˙-mediated changes in the expression of these genes.

Although the predicted sub-cellular localization of the proteins encoded by the APX genes analyzed in this report, based on the Neighbour Joining tree generated from ClustalX alignment of the maize APX proteins with Arabidopsis APX proteins of known localization, appears to agree with those predicted previously^18^, it will be important in the near future to study the localization of the proteins *in vivo* through the use of such approaches as GFP tagging because the prediction based merely on sequence similarity may not be accurate. It is worth noting, though, that the use of TargetP (http://www.cbs.dtu.dk/services/TargetP/) agrees with the Neighbour Joining prediction in this report (TargetP result not shown). Since subcellular localization may shed light on whether or not the localization of the proteins changes in response to perturbations of NO˙ content, which may advance our understanding of the functioning of APX in response to NO˙.

It is clear that, similarly to APX, the expression of BADH and CAT genes requires NO˙ and that NOS-like activity has a role in the NO˙ production. For BADH, the transcriptional suppression of BADH expression by the L-NAME treatment offers justification for the previously observed reduction in the glycine betaine content in maize leaves upon treatment of maize seedlings with L-NAME^14^. This, however, does not exclude the possibility of post-translational regulation of BADH proteins by such mechanisms as S-nitrosylation. This is even more pertinent for CAT because, despite the clear transcriptional regulation of CAT, treatment with L-NAME not only alters the intensity (and thus activity) of the CAT isoforms but also their migration pattern and this may be suggestive of a possibility that NO˙ may be required for post-translational modification of CAT proteins that in turn influences their enzymatic activities. Further experimentation is required to establish the nature of such post-translation activities beyond those observed previously^16^.

We thus conclude that NOS-like activity contributes significantly to the levels of NO˙ in maize leaves, which regulates the expression of APX, catalase and BADH. Given the role of the ROS-scavenging enzymes and glycine betaine in plant responses to stress, the results reported here emphasize the importance of NO˙ as a molecule pivotal in plant signaling and behavior with a potential to have impact of crop production under stress conditions. Importantly, given that both glycine betaine and ROS are key molecules involved in plant responses to major abiotic stresses such as drought and salinity, these results provide further understanding of how NO˙ controls the production of glycine betaine and ROS, which can be useful in developing plants with improved tolerance to these stresses through regulation of NOS-like activity.

## Materials and Methods

### Plant growth

Maize [*Zea mays* (L.) cv. Border King] seeds were surface-sterilized in 0.35% sodium hypochlorite for 10 min and then rinsed five times with sterile distilled water. The seeds were imbibed in sterile distilled water for 16 hours. After germination on wet germination paper, when the seeds had radicles of approximately 5 mm, the seeds were sown in 6 L of moist Promix Organic (Windell Hydroponics, Cape Town, South Africa) in 25 cm diameter plastic pots. Germinated seedlings (one plant per pot) were grown at a 25/19 °C day/night temperature and a 16/8 h light/dark cycle, at a photosynthetic photon flux density of 400 μmol photons.m^−2^.s^−1^ during the day phase. The Promix Organic was kept moist by irrigating the plants with 100 ml of a nutrient solution (Nitrosol®) made up in 10 mM Tris-HCl (pH 7.3) according to the manufacturer’s instructions [Fleuron (Pty) Ltd, Gardenview, South Africa] every 48 hours.

### Treatment of plants

Plants at the V1 stage of vegetative growth (when only one true leaf had a visible collar), which were of similar height, were selected for all experiments. For each treatment/untreated control, three plants were selected and each of the experiments described below were repeated three times. Plants were supplied with 100 ml of nutrient solution (untreated control) for two weeks at 48 hour intervals. For another set of plants, 100 ml of the nutrient solution was supplemented with L-NAME at a final concentration of 1 mM for the ‘L-NAME’ treatment at 48 hour intervals for two weeks. Another set of plants was supplied with 100 ml of the nutrient solution which was supplemented with a combination of both L-NAME and DETA/NO (the ‘L-NAME + DETA/NO’ treatment) at a final concentration of 1 mM for L-NAME and 200 μM for DETA/NO for two weeks at intervals of 48 hours. At the end of the two weeks of the treatment, the first and second youngest leaves from each of the plants in the three pots for each treatment were cut with a pair of scissors and placed in a 50 ml conical plastic tube and snap-frozen in liquid nitrogen. The plant material was ground to a fine powder using a mortar and pestle, returned to the conical tube and stored at −80 °C until further use.

### Measurement of nitric oxide and H_2_O_2_ content

To evaluate the effect of the ‘L-NAME’ and the ‘L-NAME + DETA/NO’ treatments on the level of NO˙ in leaf tissue, 100 mg of the maize leaf material from each treatment was homogenized in an extraction buffer containing 0.1 M sodium acetate, 1 M NaCl and 1% (w/v) ascorbate, pH 6.0. The NO˙ content was measured by following the oxyhemoglobin-based spectrophotometric assay for NO^37^.

In order to determine if the inhibition of NOS activity affects ROS accumulation, we measured H_**2**_O_**2**_ content in the maize leaf samples. The samples (100 mg from each treatment) were homogenized in 400 μl of cold 6% (w/v) trichloroacetic acid (TCA). The H_**2**_O_**2**_ content was determined as spectrophotometrically^38^.

### Identification of APX, BADH and CAT genes

APX-encoding genes were identified from Phytozome (http://phytozome.jgi.doe.gov/pz/portal) on the basis of data on maize APX genes^18^ and the homology of their corresponding encoded proteins to Arabidopsis APX proteins that have been characterized previously^39^. To complement the prediction of subcellular localization of the subset of maize APX proteins made in Liu *et al*.^18^), encoded by the genes reported here, the maize proteins were aligned to the Arabidopsis proteins using Clustal X^40^. The probable subcellular localization of the maize proteins was then assigned on the basis of a Clustal X-dependent neighbor-joining tree derived using NJPlot^41^. A keyword search using ‘betaine aldehyde dehydrogenase’ on Phytozome against the maize database was performed to identify genes potentially encoding BADH. For identification of catalase genes, a keyword search using ‘catalase’ on Phytozome against the maize database was undertaken.

### Total RNA isolation and first stand cDNA synthesis

Total RNA was extracted from the maize leaf samples (50 mg each) using the Direct-Zol™ RNA miniprep kit (Zymo Research) according to the instructions from the manufacturer. RNase-free DNase I (Zymo Research) was used to remove DNA from the isolated RNA as specified by the manufacturer. RiboLock^®^ RNase Inhibitor (Thermo Scientific) was added to each RNA sample to prevent RNase-mediated degradation of the RNA. First strand cDNA synthesis was carried out using 500 ng of total RNA and the RevertAid™ Reverse Transcriptase kit, with an Oligo(dT) 18 Primer (Thermo Scientific) as specified by the manufacturer.

### Quantitative real-time PCR analysis

Measurement of changes in BADH1, BADH2, APX6, APX4, APX3, APX1.2, CAT1 and CAT2 transcript levels in response to the inhibition of NOS-like activity was carried out using quantitative PCR (qPCR). The qPCR experiments for the untreated control, L-NAME and L-NAME + DETA/NO treatments were done in triplicate for each experiment using Luminaris Color HiGreen™ Low ROX qPCR master mix (Thermo Scientific) on the cDNA isolated as described above, following the manufacturer’s recommendations. The sequences of the primers for the experimental and the three internal control genes used were designed using Primer 3 software^42^ and are provided in Table 1. Transcript accumulation levels were expressed as ratios relative to the values of the control samples, with the transcript average accumulation levels of the internal control genes (Elongation Factor 1α, β-tubulin and Actin2) as the reference genes, nased on the 2^−ΔΔ^T method^43^.

**Table 1:** Sequences of primers used in qPCR for analysis of gene expression.

Gene Name	Forward Primer (5′–3′)	Reverse Primer (5′–3′)
APX1.2	GGCAAGCAGATGGGTTTGA	CTCCACAAGAGGGCGGAAGA
APX3	AGAAGCACCCCAAGATCACATACGC	TTTCAAGAGCCCTTCAGAGTCGC
APX4	CACTCCAACTCTCCGATCTCCAGGA	TCGAACCATTTGCACCACCAGTTTT
APX6	GAAACTTCCTGATGCTGGCCCAAGT	TTCAACTGTCCATGATTGCCCACCA
BADH1	ATTGGGGTTGTTGGACTGATCACTC	TGGGAATGTGAGGATAATGGAGCAC
BADH2	TGTGGGAAGCCTTATGATGAAGCTG	AGTTCCAAGGAGTTATCAGCCCAAC
CAT1	TCAAGCCGAATCCAAAGACCA	TCGAGCAAGCATTTCACACCA
CAT2	GCACACGTACACGCTCGTCAG	GTCTTCCATCTCGGGGTCCAT
Elongation Factor 1α	TGGGCCTACTGGTCTTACTACTGA	ACATACCCACGCTTCAGATCCT
β-tubulin	CTACCTCACGGCATCTGCTATGT	GTCACACACACTCGACTTCACG
Actin2	CTGAGGTTCTATTCCAGCCATCC	CCACCACTGAGGACAACATTACC

### Determination of protein concentration and catalase enzymatic activities

For protein extraction, 200 mg of leaf material from each treatment (in triplicate) were ground into fine powder in liquid nitrogen. The material was added to tubes containing 600 µl of extraction buffer [40 mM K_2_HPO_4_, pH7.4, 1 mM EDTA, 5% (w/v) polyvinylpolypyrrolidone (PVPP, molecular weight = 40 000) and 2 mM ascorbate]. The mixture was subjected to centrifugation at 4 °C for 10 minutes and the supernatant was taken as crude protein extract for protein concentration determination and enzymatic assays. Protein concentrations for all assays were measured in the extracts as instructed for the RC DC™ Protein Assay Kit II (Bio-Rad Laboratories).

Catalase activity was determined spectrophotometrically using a modified method of based on decomposition of H_2_O_2_^44^. For this assay, a mixture of 50 mM K_2_HPO_4_ buffer (pH 7.0) containing 0.5 mM EDTA and 50 µg of crude protein extract was prepared. The catalase reaction was initiated by addition of 30 mM H_2_O_2_ and absorbance was measured immediately. The decomposition of H_2_O_2_ was followed on the basis of the decrease in absorbance at 240 nm. The extinction coefficient of H_2_O_2_ (43.6 M^−1^ cm^−1^ at 240 nm) was used to calculate the enzymatic activity. To determine CAT activity using native polyacrylamide gel electrophoresis, native PAGE [made of 7.5% (v/v) resolving gel and 5% stacking gel] with a thickness of 1.5 mm was prepared. The gel was equilibrated with a running buffer containing 192 mM glycine, and 24 mM Tris (pH 7.2). Protein extracts (100 µg each) were loaded onto the native PAGE gel. After electrophoresis, the gel was washed with distilled water 3 times for 10 min in shaker and stained^45^. When achromatic bands formed, the stain was discarded and the gel rinsed with water and photographed.

### Statistical analyses

Data, generated from three independent experiments as described in the section ‘Materials and Methods’, were analyzed using two-way analysis of variance (ANOVA) upon which they were tested for significance by the Tukey-Kramer test at a 5% level of significance, using GraphPad Prism 6.0 software.

## Data Availability

Data reported from the study are available from the corresponding author upon request.
